# Mitochondrial dysfunction and immune microenvironment in gestational diabetes mellitus: insights from bioinformatics analysis and experimental validation

**DOI:** 10.3389/fimmu.2026.1771616

**Published:** 2026-02-26

**Authors:** Rui Zhao, Tingting Chai, Qin Gao, Aimin Jiang

**Affiliations:** 1 Department of Clinical Nutrition, The First Affiliated Hospital of Shandong First Medical University & Shandong Provincial Qianfoshan Hospital, Jinan, Shandong, China; 2 Department of Obstetrics, Jinan Maternal and Child Health Care Hospital, Jinan, Shandong, China; 3 Department of Public Health, Jining Medical University, Jining, Shandong, China; 4 Shandong Provincial Key Laboratory of Precision Oncology, Shandong Cancer Hospital and Institute, Shandong First Medical University and Shandong Academy of Medical Sciences, Jinan, Shandong, China

**Keywords:** bioinformatics analysis, gestational diabetes mellitus, immune infiltration, inflammation, mitochondrial dysfunction, placenta

## Abstract

**Background:**

Gestational diabetes mellitus (GDM) is a pregnancy−related disorder characterized by inflammatory dysregulation that disrupts maternal–fetal immune homeostasis, yet the contribution of mitochondrial dysfunction to this pro−inflammatory state remains incompletely understood.

**Methods:**

This study combined transcriptomic data obtained from the GEO repository and mitochondrial gene lists from MitoCarta3.0 to pinpoint mitochondrial-related genes (Mito-RGs) exhibiting differential expression in GDM. Machine learning algorithms, including the least absolute shrinkage and selection operator (LASSO), random forest (RF), and extreme gradient boosting (XGBoost), were applied to identify hub Mito-RGs. Gene set variation analysis (GSVA) and gene set enrichment analysis (GSEA) were performed to identify enriched pathways in various cell types. A predictive nomogram for GDM was developed based on Mito-RGs scores. Experimental validation was conducted in human placental tissues and a GDM mouse model to confirm hub gene expression.

**Results:**

DHRS2, STX17, and TIMM44 were identified as hub Mito-RGs involved in GDM. Scores based on these genes formed the basis of a nomogram with strong predictive performance for GDM. Single-cell RNA sequencing data indicated that GDM placental tissues exhibited higher proportions of epithelial cells, macrophages, and NK cells, alongside a significant reduction in tissue stem cells. Glycolysis and hypoxia-related pathways were enriched in epithelial and stem cells, whereas inflammatory and immune−activation pathways were predominantly enriched in macrophages, indicating pro−inflammatory remodeling of the placental immune microenvironment. Immunohistochemistry confirmed significantly elevated DHRS2 protein levels in placentas from GDM patients and GDM mouse models.

**Conclusions:**

These findings emphasize the critical impact of mitochondrial dysfunction on the pro−inflammatory reprogramming of the placental immune microenvironment in GDM, providing potential targets for anti−inflammatory and immunometabolic interventions.

## Introduction

Gestational diabetes mellitus (GDM), characterized by the onset or initial recognition of hyperglycemia during pregnancy, is a common complication affecting about 14% of pregnancies globally (1). GDM not only compromises maternal health, increasing the risk of cesarean delivery, type 2 diabetes mellitus (T2DM), and cardiovascular disease, but also leads to significant adverse outcomes for the fetus, such as macrosomia, neonatal hypoglycemia, and long-term metabolic disorders (2–4). Despite the substantial public health burden posed by GDM, the mechanisms underlying its progression remain incompletely understood. This highlights the critical need for additional research into its pathogenesis and the discovery of reliable biomarkers for early diagnosis.

Mitochondria are intricate double-membrane organelles that, in addition to producing adenosine triphosphate (ATP) through oxidative phosphorylation, are also indispensable for calcium regulation, reactive oxygen species (ROS) production, immune signaling, and apoptosis (5). Mitochondrial dysfunction is linked to the development of chronic diseases such as diabetes, cardiovascular disease, and metabolic syndromes (6–8). Emerging evidence indicates that mitochondrial dysfunction plays a crucial role in the pathological changes of GDM by disrupting metabolic signaling and promoting oxidative stress within the placenta, a vital maternal-fetal exchange site (9–11). For instance, a study found a notable decrease in mitochondrial complex expression *in vivo* and impaired mitochondrial respiration *in vitro* in placentas from GDM pregnancies (12). Additionally, GDM patients exhibited markedly elevated mitochondrial DNA (mtDNA) levels in maternal circulation compared to healthy controls (13). Furthermore, Qiu et al. demonstrated that placental mtDNA copy number is significantly and positively associated with increased oxidative stress in GDM pregnancies (14). Although previous studies have demonstrated the critical role of mitochondrial dysfunction in the pathophysiology of GDM, few have explored mitochondrial-related biomarkers for GDM using integrated bioinformatics approaches. Therefore, investigating the role of mitochondrial-related genes (Mito-RGs) in GDM is essential, providing important insights for early diagnosis and effective treatment strategies.

Emerging evidence indicates that immune dysregulation is pivotal to the development of GDM (15). In GDM placental tissues, altered immune cell infiltration has been observed, including dysregulated macrophages, neutrophils, and T-cell subsets (16–18). These immune cells interact closely with trophoblasts, creating a pro-inflammatory environment that promotes insulin resistance (19). Importantly, mitochondrial dysfunction and immune dysregulation appear to be closely intertwined, forming a vicious cycle. Therefore, further exploration of the unique immune microenvironment in GDM and its interaction with mitochondrial dysfunction is crucial for unraveling the complex pathology of GDM.

This study provides significant insights into the role of Mito-RGs as potential diagnostic biomarkers for GDM and characterizes the unique immune microenvironment interactions with mitochondrial dysfunction. By utilizing RNA sequencing (RNA-*seq*) data alongside multiple machine learning algorithms, we systematically identified hub Mito-RGs. Furthermore, our analysis of single-cell RNA-*seq* (scRNA-*seq*) data enables the precise characterization of the immune microenvironment in GDM patients at a single-cell level. Additionally, we performed experimental validation of the identified Mito-RGs by collecting placental tissues from GDM patients and establishing a GDM mouse model, laying the groundwork for future research aimed at unraveling the complex pathophysiology of GDM and identifying therapeutic targets.

## Materials and methods

### Data acquisition

Gene expression datasets for GDM and healthy pregnancies were obtained from the Gene Expression Omnibus (GEO) database (https://www.ncbi.nlm.nih.gov/geo). The bulk RNA-*seq* dataset (GSE203346) includes data from 21 GDM patients and 22 healthy pregnant women (**Supplementary Table 1**) (20). Moreover, scRNA-*seq* data (GSE173193) from placental tissue samples of two GDM patients and two healthy pregnant women were utilized (**Supplementary Table 2**) (21). A comprehensive list of 1, 136 Mito-RGs was extracted from the MitoCarta3.0 database (22) (https://www.broadinstitute.org/mitocarta).

### Identification of differentially expressed Mito-RGs and functional enrichment analysis

Differentially expressed Mito-RGs between the GDM and control groups were identified using the R package “limma” (version 3.65.1) on bulk RNA-*seq* data, with the criteria set at |log2FoldChange (FC)| > 0.585 and adjusted *P*-value < 0.05. Volcano plots and heatmaps were used to visualize the results by the R packages “ggplot2” (version 3.5.2) and “pheatmap” (version 1.0.12). The biological functions of Mito-RGs were further clarified through Gene Ontology (GO) and Kyoto Encyclopedia of Genes and Genomes (KEGG) enrichment analyses, utilizing the R package “clusterProfiler” (version 4.14.4). GO analysis offered functional annotation for enriched differential genes across biological processes, cellular components, and molecular functions. KEGG pathway analysis utilized a hypergeometric test to identify the genetic functions based on gene enrichment in specific pathways. Gene set enrichment analysis (GSEA) was performed to detect pathways associated with mitochondria. Genes were ranked by log2FC, with pathways deemed significant if their normalized enrichment score (NES) > 1 or < -1, and their *P*-value was less than 0.05.

### Identification of hub Mito-RGs by machine learning algorithms

Three machine learning algorithms—least absolute shrinkage and selection operator (LASSO) regression, random forest (RF), and extreme gradient boosting (XGBoost)—were employed to identify Hub Mito-RGs. The R package “glmnet” (version 4.1.8) was utilized to conduct LASSO regression, identifying genes with non-zero coefficients by optimizing lambda parameters via 10-fold cross-validation. The RF algorithm was applied using the R package “RandomForest” (version 4.7-1.2) to estimate and rank the importance of Mito-RGs, with the top 10 genes exhibiting the highest MeanDecreaseAccuracy scores selected for further analysis. For XGBoost, the R package “xgboost” (version 1.7.9.1) was utilized to iteratively optimize feature weights, with hyperparameter tuning implemented through grid search combined with 10-fold cross-validation to ensure robust performance. The top 10 genes ranked by Gain importance scores were extracted as candidate features. The genes identified by all three algorithms were designated as hub Mito-RGs, ensuring reliable identification of key Mito-RGs associated with GDM.

### Establishment and validation of a GDM prediction model based on Mito-RGs score

The Mito-RGs scores were calculated using multivariate logistic regression, with gene expression levels weighted by their regression coefficients and standardized *via* z-score normalization. Patients were divided into high-score (z-score > 0) and low-score (z-score ≤ 0) categories. Spearman correlation analysis was conducted to evaluate relationships between Mito-RGs scores and clinical variables, including maternal age, body mass index (BMI), oral glucose tolerance test (OGTT) results, and gestational age. A nomogram was developed using the R package “rms” (version 6.9.0), incorporating Mito-RGs scores alongside clinical parameters. The discriminatory ability of the nomogram was evaluated by calculating the area under the ROC curve (AUC). Calibration curves and decision curve analysis (DCA) were employed to evaluate its calibration accuracy and clinical utility in predicting GDM risk.

### Immune cells infiltration landscape of GDM

The ImmuCellAI algorithm was used to assess the infiltration levels of different immune cell types in placental tissues from both GDM and healthy controls (23). This tool analyzed bulk RNA-*seq* transcriptional profiles to determine the proportions of immune cell subsets, including T cells, B cells, macrophages, dendritic cells (DCs), and natural killer (NK) cells, in the placental microenvironment. Immune cell infiltration differences between the GDM and control groups were quantified using log2FC for immune cell proportions. A positive log2FC indicated increased infiltration in the GDM group, whereas a negative log2FC reflected reduced infiltration compared to controls. The R package “pheatmap” was utilized to create a heatmap, illustrating the immune cell infiltration landscape and emphasizing variations in cell type abundance.

### scRNA-*seq* data analysis

For scRNA-*seq* data analysis, initial quality control (QC) was conducted to filter out cells with fewer than 100 expressed genes or those with over 10% mitochondrial gene expression, ensuring high-quality data. Expression matrices were normalized using log-normalization, and highly variable genes were selected for downstream analyses. Dimensionality reduction was performed using principal component analysis (PCA), and graph-based clustering was subsequently conducted with the R package “Seurat” (version 5.1.0). Clusters were visualized in low-dimensional space using the uniform manifold approximation and projection (UMAP). Cell type annotation was performed with SingleR (version 2.8.0) by matching cluster expression profiles against well-established reference datasets. Expression levels of hub genes were visualized across the identified clusters using feature plots and violin plots, showing their specific abundance patterns. The Mito-RGs score at the single-cell level was calculated using the “AddModuleScore” function in Seurat, allowing aggregated expression values to be mapped onto the UMAP projection for spatial distribution analysis. The Mito-RGs scores were mapped onto the UMAP projection through feature plots to reveal their spatial distribution across cell subtypes. Biological functional differences across cell types were examined using gene set variation analysis (GSVA) enrichment analysis and single-cell GSEA (24) with predefined hallmark gene sets. Additionally, the “CellChat” package (version 2.2.0) was applied to infer intercellular communication networks based on ligand-receptor interactions, with a focus on mitochondrial-related pathways. Ligand-receptor pairs mediating key biological functions were identified, and interaction intensity and directionality were visualized to uncover the underlying cellular communication patterns.

### Collection of human placental tissues

Placental tissues were collected from pregnant women with GDM and corresponding healthy controls. All samples were obtained from a teaching hospital affiliated with the Shandong First Medical University. GDM was diagnosed based on the International Association of Diabetes and Pregnancy Study Groups (IADPSG) criteria, which include fasting blood glucose (FBG) levels of at least 5.1 mmol/L, 1-hour postprandial glucose levels of at least 10.0 mmol/L, or 2-hour postprandial glucose levels of at least 8.5 mmol/L during OGTT. Placental tissues from the maternal-fetal interface were collected following vaginal delivery. The study adhered to the Declaration of Helsinki and received approval from the Ethics Committee of the First Affiliated Hospital of Shandong First Medical University (Approval Number: LCYJLL2024-S127).

### Establishment of the GDM murine model

The *in vivo* phase of this study, encompassing all animal experimentation and tissue collection, was carried out over a five-month period from March 15, 2024, to July 20, 2024. Female C57BL/6-J mice aged six weeks (16–18 g) were purchased from Jinan Pengyue Experimental Animal Breeding Co., Ltd. (Shandong, China). Mice were provided unrestricted access to food and water and maintained under controlled conditions: a temperature of 22 ± 1°C, relative humidity of 55 ± 1%, and a 12/12 h light/dark cycle. After one week of acclimation, they were randomly divided into a control group (n=8) and a high-fat diet (HFD) group (n=8). A GDM model was established by feeding the HFD group a diet containing 60% of calories from fat (Research Diets) to induce insulin resistance. The HFD regimen was initiated six weeks prior to mating and continued throughout the gestation period. Control mice received a low-fat diet (LFD) with 10% of calories derived from fat (Research Diets). GDM modeling was confirmed by establishing a significantly higher glucose AUC in the HFD group compared to the control group. On gestational day 18 (G18), a glucose tolerance test was performed. After a 12-hour fast, FBG levels were measured using a glucometer. A 20% glucose solution was then administered *via* oral gavage, and tail vein blood glucose concentrations were subsequently measured at 30, 60, 90, and 120 minutes post-gavage. Maternal body weight was recorded from pre-pregnancy to the end of gestation. The successful induction of GDM was confirmed by sustained hyperglycemia, insulin resistance, and distinct weight gain patterns compared to controls. After establishing the GDM model, we ensured that three mice from each group were selected for final analysis. After a 12-hour fast, all mice were euthanized between June 10 and June 25, 2024, and samples including blood and placental tissue were collected. Euthanasia was carried out under deep isoflurane anesthesia followed by cervical dislocation, consistent with the American Veterinary Medical Association (AVMA) Guidelines. All animal procedures were performed in accordance with the ARRIVE (Animal Research: Reporting *In Vivo* Experiments) guidelines and approved by the Institutional Animal Care and Use Committee of the First Affiliated Hospital of Shandong First Medical University (Approval Number: LCYJLL2024-S128).

### Immunohistochemistry staining and analysis

IHC staining was conducted to evaluate the protein expression of dehydrogenase/reductase member 2 (DHRS2), syntaxin 17 (STX17), and translocase of inner mitochondrial membrane 44 (TIMM44). Human and mouse placental tissues were fixed in 4% paraformaldehyde, dehydrated, cleared with xylene, and embedded in paraffin for histological analysis. Sections underwent deparaffinization, rehydration, and antigen retrieval using citrate buffer (pH 6.0). Endogenous peroxidase was blocked with 3% hydrogen peroxide, and nonspecific binding was reduced with 5% bovine serum albumin. Overnight incubation of tissue sections at 4 °C was performed with primary antibodies: DHRS2 (Proteintech, 15735-1-AP), STX17 (Affinity Biosciences, DF12483), and TIMM44 (Affinity Biosciences, DF12332). Sections were washed, incubated with HRP-conjugated secondary antibodies, developed using diaminobenzidine, and counterstained with hematoxylin. Protein expression was semi-quantitatively evaluated using an IHC scoring method incorporating staining intensity and the extent of positive staining. Intensity (I) was graded from 0 to 3 (0, none; 1, weak; 2, moderate; 3, strong). The proportion of positively stained cells (A) was categorized on a 1–4 scale (1, <10%; 2, 10–50%; 3, 50–90%; 4, >90%). The overall IHC score was computed as I×A.

### Statistical analysis

R software (version 4.3.1) was used for statistical analyses. Continuous variables are expressed as the mean ± standard deviation (SD) and categorical data are presented as n (%). For comparisons between two groups, the Student’s *t*-test was applied for continuous variables and the *Chi*-square test for categorical variables. Spearman’s rank correlation was used for correlation analyses. Logistic regression models were used for univariate and multivariate analyses, with results expressed as odds ratios (OR) and 95% confidence intervals (95% CI). *P*-values for multiple comparisons were adjusted using the Benjamini–Hochberg method to control the false discovery rate (FDR), with an adjusted *P*-value < 0.05 considered statistically significant.

## Results

### Differentially expressed Mito-RGs and functional enrichment analysis

**Figure 1:**
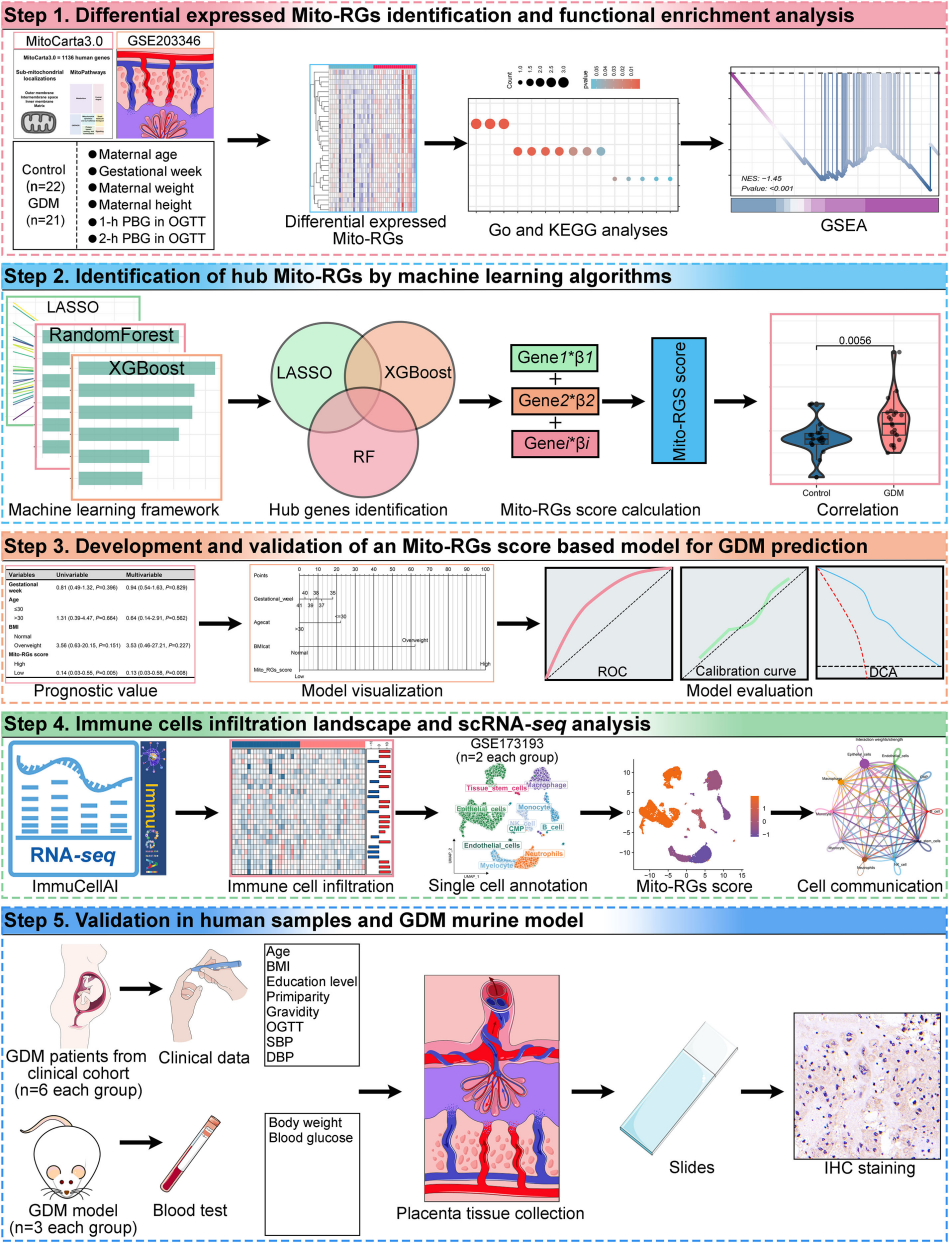
The flowchart of the overall study procedures.

**Figure 2:**
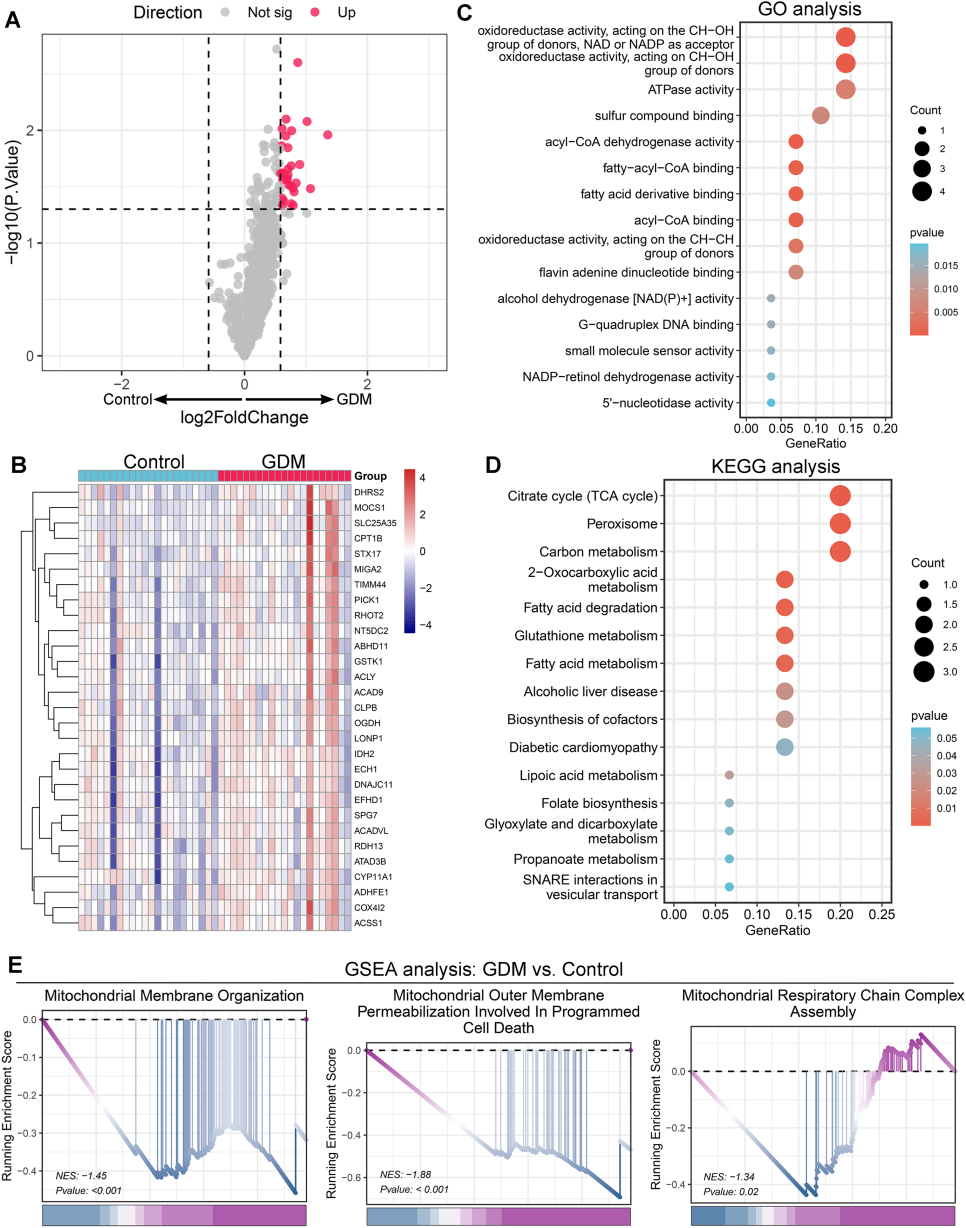
Identification and functional enrichment analysis of differentially expressed mitochondrial-related genes (Mito-RGs) in gestational diabetes mellitus (GDM). **(A)** Volcano plot showing the distribution of Mito-RGs between the GDM and control groups. Red dots indicate significantly upregulated genes, while gray dots represent non-significant genes. **(B)** Heatmap illustrating the expression patterns of Mito-RGs in the GDM and control groups. **(C)** Bubble plot displaying the Gene Ontology (GO) enrichment analysis for the identified Mito-RGs, where the bubble size reflects the gene count and the color represents the enrichment p-value. **(D)** Bubble plot showing the Kyoto Encyclopedia of Genes and Genomes (KEGG) pathway enrichment analysis. **(E)** Gene set enrichment analysis (GSEA) revealed that mitochondrial dysfunction is implicated in GDM.

The flow diagram of this study is illustrated in **Figure 1**. This study examined the gene expression dataset GSE203346, consisting of 21 placental tissue samples from women with GDM and 22 from healthy controls. By intersecting the dataset with the MitoCarta3.0 database, 29 differentially expressed Mito-RGs were identified, all of which exhibited significant upregulation in GDM samples compared to controls. The distribution of these Mito-RGs is visualized in the volcano plot (**Figure 2A**), and their expression patterns were further illustrated using a hierarchical clustering heatmap (**Figure 2B**). Functional enrichment analysis of the identified Mito-RGs revealed key molecular processes and pathways central to the pathophysiology of GDM. GO analysis revealed significant enrichment in processes like oxidoreductase activity, fatty acid binding, and small molecule metabolism (**Figure 2C**). KEGG pathway analysis identified the participation of metabolic pathways, including the citrate cycle (TCA cycle), fatty acid metabolism, and glutathione metabolism (**Figure 2D**). GSEA highlighted the downregulation of mitochondrial-related pathways in GDM, such as mitochondrial membrane organization, mitochondrial outer membrane permeabilization linked to programmed cell death, and mitochondrial respiratory chain complex assembly (**Figure 2E**).

### Hub Mito-RGs in GDM by machine learning algorithms

**Figure 3:**
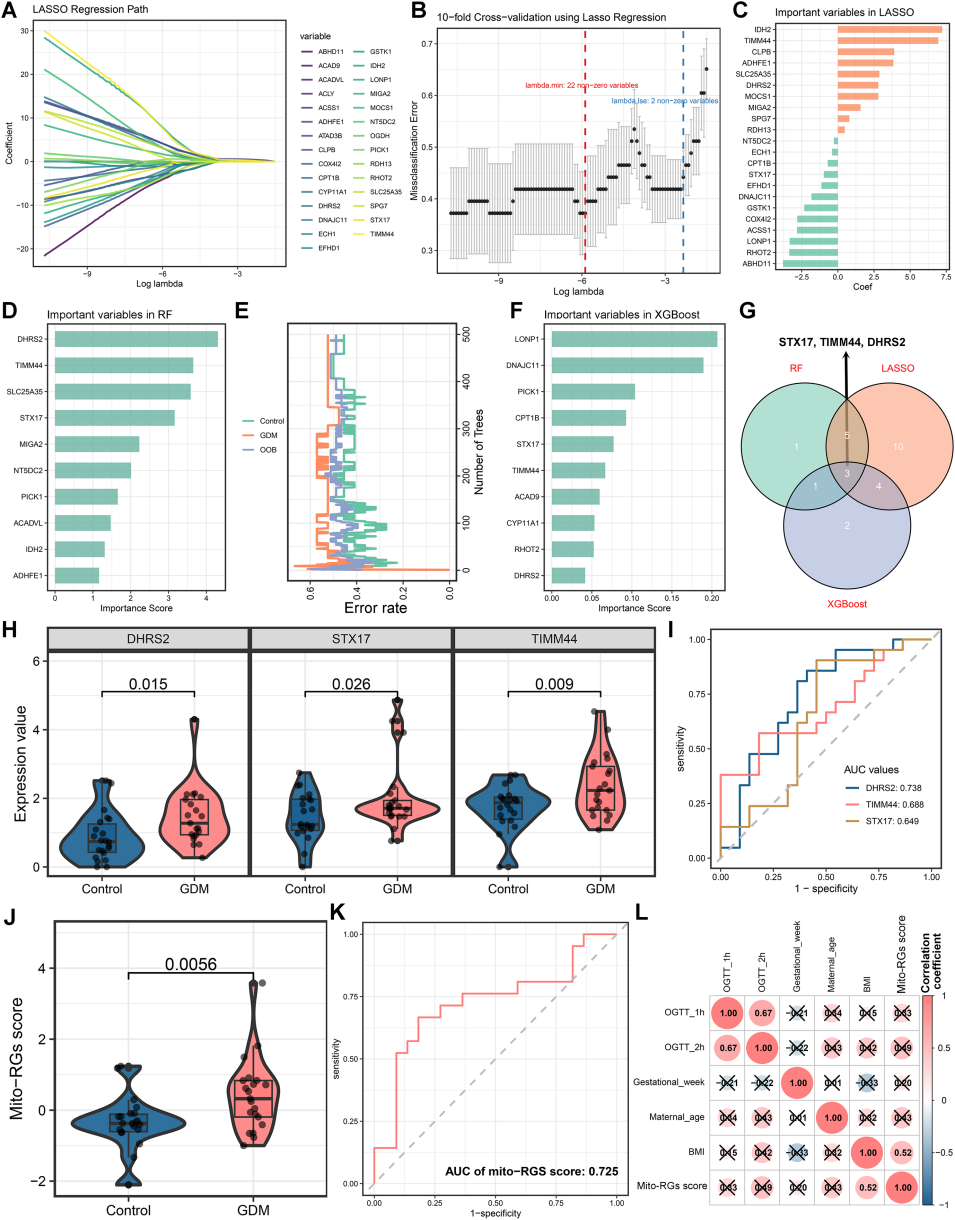
Identification of hub mitochondrial-related genes (Mito-RGs) and development of the Mito-RGs score. **(A)** Least absolute shrinkage and selection operator (LASSO) regression path plot depicting the coefficient shrinkage process for variable selection across different λ values. **(B)** Ten-fold cross-validation to determine the optimal λ in LASSO regression. **(C)** Coefficients of Mito-RGs selected by LASSO regression. **(D)** Importance ranking of variables based on the random forest (RF) model. **(E)** Relationship between the number of decision trees in the RF model and the out-of-bag (OOB) error and classification error rates. **(F)** Importance ranking of variables in the extreme gradient boosting (XGBoost) model. **(G)** Venn diagram displaying the hub Mito-RGs identified through the intersections of the three machine learning algorithms. **(H)** Comparison of mRNA expression levels of the identified hub Mito-RGs between gestational diabetes mellitus (GDM) and control placental tissues. **(I)** Receiver operating characteristic (ROC) curves of the three hub genes for GDM prediction. **(J)** Comparison of the Mito-RGs score between GDM and control groups. **(K)** ROC curve of the mito-RGS score for GDM prediction. **(L)** Correlation heatmap showing the relationships between the Mito-RGs score and clinical variables.

In the LASSO regression analysis, the coefficient shrinkage process was visualized (**Figure 3A**). Through 10-fold cross-validation, the optimal λ value was identified, leading to the selection of 22 genes from the 29 differentially expressed Mito-RGs as potential GDM biomarkers with minimal error (**Figures 3B, C**). The RF analysis ranked the variables by importance and selected the top 10 genes as potential candidate biomarkers for GDM (**Figure 3D**). The stability and classification performance of the RF model were verified by plotting the out-of-bag (OOB) error and classification error rates with an increasing number of decision trees (**Figure 3E**). In addition, the XGBoost analysis ranked the importance of the genes and identified the top 10 important contributing genes to GDM (**Figure 3F**). A Venn diagram integrating the results from all three machine learning algorithms identified DHRS2, STX17, and TIMM44 as hub Mito-RGs in GDM (**Figure 3G**). The expression levels of the three hub genes were markedly elevated in GDM placental tissues relative to controls (**Figure 3H**). ROC curve analysis demonstrated the diagnostic performance of each hub gene for GDM, yielding AUC values of 0.738 for DHRS2, 0.649 for STX17, and 0.688 for TIMM44 (**Figure 3I**). A composite Mito-RGs score, derived from the three hub genes, was significantly elevated in the GDM group relative to the controls (**Figure 3J**). Furthermore, the AUC for the composite Mito-RGs score was 0.725 (**Figure 3K**), indicating its predictive capability. Correlation analysis *via* a heatmap demonstrated positive association between the Mito-RGs score and BMI (**Figure 3L**).

### Mito-RGs score-based nomogram establishment and immune cell infiltration estimation in GDM

**Figure 4:**
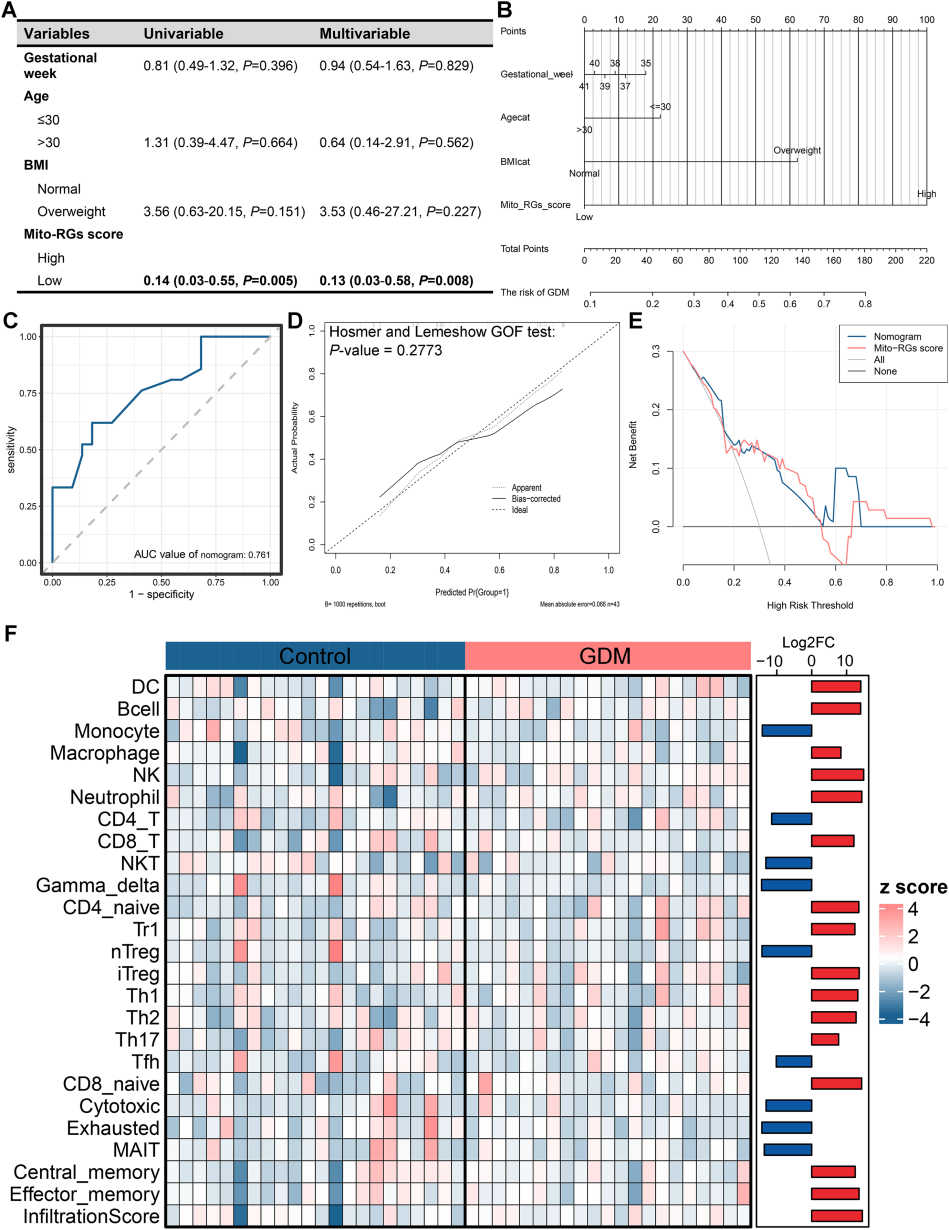
Development and evaluation of a gestational diabetes mellitus (GDM) predictive model based on the mitochondrial-related genes (Mito-RGs) score and immune infiltration analysis. **(A)** Univariate and multivariate logistic regression analyses examining the association between the Mito-RGs score and GDM. **(B)** Nomogram for predicting individual GDM risk. **(C)** Receiver operating characteristic (ROC) curve demonstrating the predictive performance of the Mito-RGs score for GDM. **(D)** Calibration curve evaluating the accuracy of the nomogram model. **(E)** Decision curve analysis (DCA) assessing the clinical utility of the nomogram model. **(F)** Heatmap comparing the abundance of immune cell infiltration in the placental tissues of GDM patients and healthy controls, analyzed using the ImmuCellAI algorithm.

**Figure 4A** illustrates the results of logistic regression analyses assessing potential predictors of GDM. In both univariate and multivariate analyses, there was a statistically significant association between Mito-RGs scores and GDM risk, suggesting that Mito-RGs scores are a strong independent predictor for GDM. To facilitate clinical application, a nomogram was constructed by integrating key variables, including gestational week, maternal age, BMI category, and the Mito-RGs score (**Figure 4B**). The nomogram allows for individualized GDM risk assessment by assigning points to each predictor variable, summing the total score, and deriving the corresponding risk probability. The ROC analysis demonstrated that the nomogram had a favorable diagnostic performance, achieving an AUC of 0.761 (**Figure 4C**). The calibration curve demonstrated strong concordance between predicted and observed GDM risk probabilities, validating the reliability of the nomogram (**Figure 4D**). DCA demonstrated that the nomogram provides substantial net clinical benefits across a wide range of risk thresholds compared to other models, particularly in the ranges of 0.08-0.22 and 0.55-0.69 (**Figure 4E**).

Immune cell infiltration differences between GDM and control placental tissues were analyzed using the ImmuCellAI algorithm. The heatmap and bar chart highlighted significant alterations in immune cell profiles in GDM samples, characterized by increased infiltration of DCs, macrophages, NK cells, and CD8^+^ T cells (**Figure 4F**). These findings suggest an abnormal activation of immune function in GDM patients, characterized by changes in placental immune cell composition.

### Validation of the hub Mito-RGs at single-cell resolution

**Figure 5:**
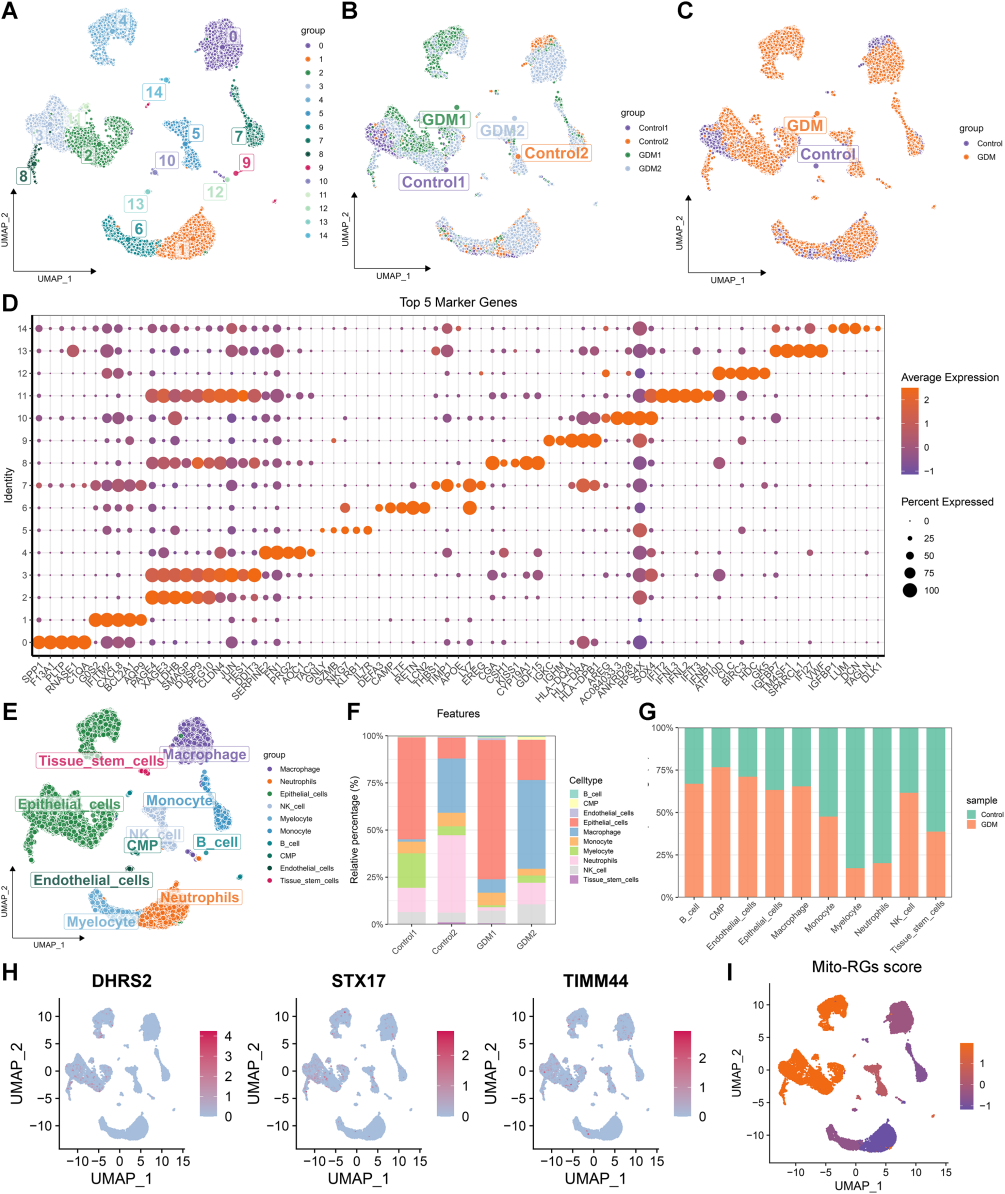
Single-cell resolution reveals the expression of hub mitochondrial-related genes (Mito-RGs) and the immune landscape in gestational diabetes mellitus (GDM). **(A)** Uniform manifold approximation and projection (UMAP) plot presenting distinct cell clusters identified from single-cell RNA sequencing (scRNA-*seq*) data. **(B)** UMAP plot displaying the distribution of cells across different individual samples, including GDM and control groups. **(C)** UMAP plot comparing cell distributions between GDM and control groups. **(D)** Bubble plot highlighting the top five marker genes for each cell cluster, aiding in the identification of major cell types. **(E)** UMAP plot with annotated cell types, including tissue stem cells, epithelial cells, macrophages, monocytes, neutrophils, natural killer (NK) cells, B cells, endothelial cells, myelocytes, and common myeloid progenitors (CMPs). **(F)** Bar plot illustrating the proportions of different identified cell types in each sample. **(G)** Bar plot comparing the proportions of major cell types between the GDM and control groups. **(H)** Feature plots depicting the expression patterns of hub Mito-RG (DHRS2, STX17, and TIMM44) in specific cell clusters. **(I)** Feature plot showing the distribution of Mito-RGs scores across various cell subpopulations, reflecting their enrichment in particular immune and stromal cell types.

scRNA-*seq* analysis was conducted to confirm the expression and localization of hub Mito-RGs in GDM. After QC (**Supplementary Figure 1**), the total number of cells analyzed included 4959 and 4366 cells from control samples, and 6114 and 4366 cells from GDM samples. The GSE173193 dataset was dimensioned into 15 distinct clusters (**Figure 5A**). Notably, a clear separation between cell populations from GDM and control samples was observed (**Figures 5B, C**), indicating substantial differences in cellular composition. Cluster-specific marker gene identification (**Figure 5D**) and SingleR-based annotation (**Figure 5E**) delineated major cell populations, including epithelial cells, endothelial cells, monocytes, macrophages, neutrophils, myelocytes, NK cells, B cells, common myeloid progenitors (CMPs), and tissue stem cells. Quantitative analysis suggested altered cell-type proportions in the GDM group compared with controls, with a trend toward higher proportions of epithelial cells, macrophages, NK cells, and B cells and a lower proportion of tissue stem cells (**Figures 5F, G**), although substantial inter-individual variability was observed. Feature plots illustrated DHRS2, STX17, and TIMM44 were predominantly expressed in epithelial cells, macrophages, tissue stem cells, and NK cells (**Figure 5H**). Moreover, the Mito-RGs score was mapped across cell subtypes, showing pronounced enrichment in epithelial cells, tissue stem cells, NK cells, and macrophages (**Figure 5I**).

**Figure 6:**
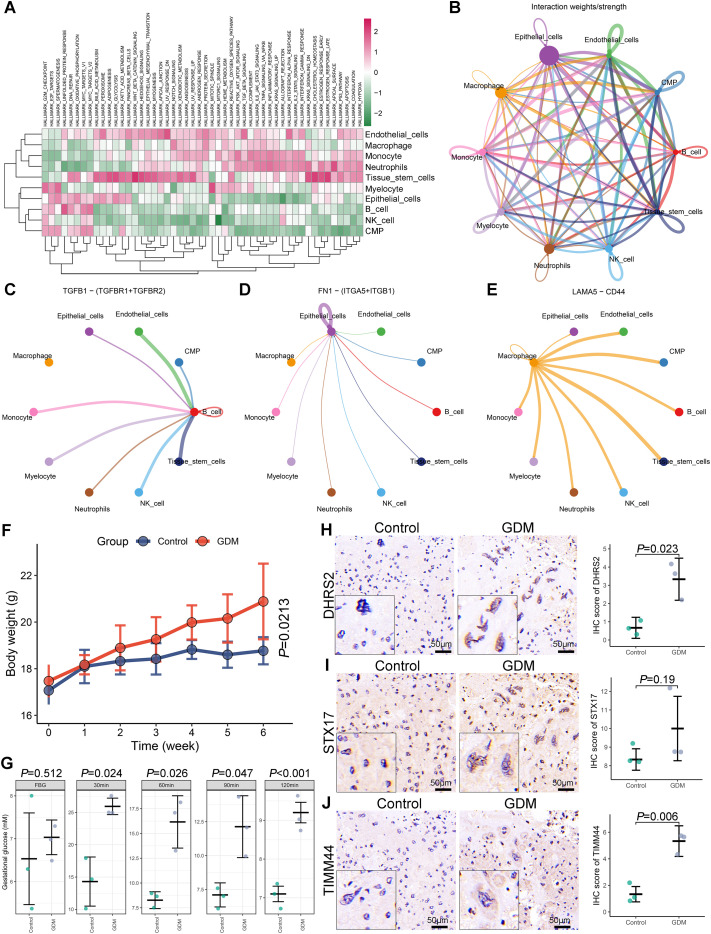
Cell-cell communication analysis and experimental validation of hub mitochondrial-related genes (Mito-RGs) expression. **(A)** Heatmap of gene set variation analysis (GSVA) enrichment across different cell subtypes. **(B)** Cell-cell communication network diagram illustrating interactions among various cell subtypes. **(C-E)** Ligand-receptor pair analysis of key signaling pathways: **(C)** TGFB1-TGFBR1/TGFBR2, **(D)** FN1-ITGA5/ITGB1, and **(E)** LAMA5-CD44. **(F)** Comparison of body weight changes between gestational diabetes mellitus (GDM) and control mice. **(G)** Comparison of blood glucose levels between GDM and control mice at different time points during the oral glucose tolerance test (OGTT). **(H-J)** Immunohistochemistry (IHC) staining and scoring of DHRS2 **(H)**, STX17 **(I)**, and TIMM44 **(J)** in placental tissues from GDM and control mice.

Single-cell GSVA enrichment analysis indicated notable activation of lipid metabolism, glycolysis, oxidative phosphorylation, and proliferation pathways in epithelial cells. Similarly, macrophages exhibited activation in apoptosis, inflammatory responses, and metabolic pathways. Meanwhile, biological processes related to proliferation were significantly activated in NK cells (**Figure 6A**). The results of single-cell GSEA further confirmed that glycolysis, oxidative phosphorylation, hypoxia, apoptosis, and reactive oxygen species-related pathways were predominantly activated in epithelial cells (**Supplementary Figures 2A, B**). Meanwhile, multiple inflammatory activation-related pathways, including antigen processing and presentation, IL6-JAK-STAT3 signaling pathway, inflammatory response, and TNF-α signaling *via* NF-κB were predominantly enriched in macrophages (**Supplementary Figure 2C**). The cell-cell communication network analysis highlighted strong interactions among epithelial cells, macrophages, and monocytes in GDM (**Figure 6B**). Key ligand-receptor pathways were identified, including TGFB1-TGFBR1/TGFBR2 signaling between B cells and other cell types (**Figure 6C**), FN1-ITGA5/ITGB1 signaling between epithelial and other cells (**Figure 6D**), and LAMA5-CD44 signaling between macrophages and other cell types (**Figure 6E**).

### Validation of hub Mito-RGs in human placental tissues and a GDM mouse model

A total of 12 pregnant women (mean age: 29.73 ± 1.07) were finally included in our study and divided into the GDM group (n = 6) and control group (n = 6). The clinical characteristics of patients are summarized in **Supplementary Table 3**. To validate the findings, human placental tissues were analyzed *via* IHC analysis. The study found a notable upregulation in DHRS2 and STX17 expression in placental samples from women with GDM compared to controls, whereas TIMM44 levels showed no significant difference between the groups (**Supplementary Figures 3A–C**).

A GDM mouse model was developed to further validate these findings. Body weight monitoring showed consistently higher weights in GDM mice compared to controls, with statistical significance reached by week 6 (*P*-value = 0.0213; **Figure 6F**). OGTT results revealed significantly elevated blood glucose levels in GDM mice at 30, 60, 90, and 120 minutes post-glucose administration compared to controls, while FBG levels showed no significant difference (**Figure 6G**). IHC analysis of murine placental tissues further validated the upregulation of hub Mito-RGs. In GDM placentas, DHRS2 and TIMM44 expression levels were significantly elevated compared to controls, while STX17 exhibited an upward trend that did not achieve statistical significance (**Figures 6H–J**).

## Discussion

Mitochondrial dysfunction has been increasingly recognized as a central driver of metabolic diseases, including GDM (6–10). In line with previous reports (9, 25–27), our transcriptomic analysis revealed significant upregulation of Mito-RGs in GDM placental tissues. Functional enrichment analyses revealed that these genes primarily participate in essential energy metabolism pathways, including oxidative phosphorylation, fatty acid metabolism, and the TCA cycle. Notably, GSEA revealed suppression of pathways related to mitochondrial structure and ATP generation, supporting the notion that impaired mitochondrial function is a hallmark of GDM placentas. These findings are consistent with earlier studies demonstrating reduced mitochondrial bioenergetics, diminished fusion marker expression, and excess ROS production, which contribute to pathological adaptations and worse fetal outcomes in GDM (28, 29).

Through transcriptomic analysis, we identified three hub Mito-RGs—DHRS2, TX17, and TIMM44—that were significantly associated with GDM. STX17 plays a critical role in autophagy, specifically mitophagy, by facilitating the removal of damaged mitochondria to maintain cellular homeostasis (30). Dysregulated STX17-mediated autophagy disrupts cellular balance, leading to organelle accumulation, oxidative stress, and inflammatory activation (31, 32). Moreover, elevated STX17 expression induces mitochondrial Ca^2+^ overload and excessive ROS production, triggering mitochondrial dysfunction (33). Silencing STX17 mitigates mitochondrial degradation, lowers ROS levels, promotes endothelial nitric oxide synthase (eNOS) phosphorylation, and diminishes apoptosis (34). TIMM44, part of the import motor within the inner mitochondrial membrane translocase complex, is crucial for mitochondrial protein import and assembly (35). Dysregulation of TIMM44 impairs bioenergetics and protein homeostasis, aggravating mitochondrial dysfunction. Similarly, DHRS2 is critical for maintaining redox balance and mitigating oxidative stress by preventing excessive ROS production (36). The function is essential for sustaining mitochondrial integrity and efficient energy metabolism. These findings offer valuable insights into the pivotal role of mitochondrial dysfunction in GDM pathophysiology and highlight potential Mito-RGs, including DHRS2, STX17, and TIMM44, as promising biomarkers for future research and therapeutic interventions. To validate our findings, we assessed gene expression in both human and mouse GDM placentas. DHRS2 and STX17 were upregulated in human GDM placentas, whereas DHRS2 and TIMM44 were elevated in the GDM mouse model. Notably, DHRS2 was consistently upregulated across bioinformatics, human, and mouse data, suggesting it may be a key regulator in GDM. Discrepancies in STX17 and TIMM44 expression between human and mouse tissues may result from species-specific regulation, sample heterogeneity, or methodological limitations (37). Thus, future studies with larger cohorts and more quantitative approaches are needed to clarify their roles and potential as clinical biomarkers in GDM.

Recent research indicates that the composition and function of the immune microenvironment are essential in GDM development and progression (15). This study found a notable increase in pro-inflammatory immune cells, such as macrophages, NK cells, DCs, and CD8^+^ T cells, in GDM placental tissues compared to healthy controls, consistent with previous reports (18, 38). These results indicate that immune overactivation contributes to the pathophysiology of GDM. To further characterize these changes, scRNA-*seq* analysis revealed elevated proportions of epithelial cells, macrophages, and NK cells, and a marked reduction in tissue stem cells in GDM placentas. At the single-cell level, DHRS2 expression was mainly localized to epithelial cells and tissue stem cells, whereas STX17 and TIMM44 were also expressed in macrophages and NK cells. These findings suggest that mitochondrial dysfunction in these specific placental cell types may underlie the altered immune landscape of GDM. Further pathway analyses confirmed significant activation of mitochondrial dysfunction–related biological processes—including glycolysis, hypoxia, ROS production, and oxidative phosphorylation—in epithelial cells and tissue stem cells from GDM placentas. This aligns with published data showing that mitochondrial disturbances in trophoblasts, the predominant epithelial cell type, contribute to placental dysfunction and GDM via mechanisms such as elevated ROS and HIF-1α activation (39). Notably, we also observed upregulation of key inflammatory pathways, such as IL6-JAK-STAT3 and TNF-α signaling, in macrophages, indicating a shift toward a pro-inflammatory phenotype, as previously described by Chen et al. (38). The reduction in tissue stem cell populations, together with clear evidence of mitochondrial dysfunction, suggests that strategies aimed at restoring mitochondrial function could hold therapeutic promise for GDM. In summary, these results indicate that mitochondrial dysfunction in epithelial cells, tissue stem cells, and macrophages contributes to the formation of a pro-inflammatory immune microenvironment in GDM. Further research is warranted to clarify the cell type-specific roles of the identified hub genes, which may provide new targets for therapeutic intervention and risk prediction in GDM.

A major strength of this study is the application of multiple machine learning algorithms, including LASSO, RF, and XGBoost, to effectively prioritize Mito-RGs associated with GDM. This approach enabled the robust identification of DHRS2, STX17, and TIMM44 as hub genes from a large pool of candidates. These hub genes demonstrated significant predictive power in distinguishing GDM from healthy controls, and the expression was further validated *via* IHC staining of both human and mouse placental tissues. The integration of bioinformatics analysis with experimental validation enhances the reliability and robustness of our results. Additionally, developing the Mito-RGs score, based on these hub genes, introduces an innovative approach for GDM prediction. Incorporating the Mito-RGs score into a nomogram alongside clinical variables significantly enhanced the predictive performance and practicality of the model. With the rising prevalence of GDM, such predictive tools offer valuable potential for earlier diagnosis and precise stratification of high-risk pregnancies, paving the way for more targeted and effective interventions to improve maternal and fetal outcomes.

However, this study has several limitations. Firstly, a key limitation of our study is the small sample size, especially concerning the scRNA-*seq* analysis. The limited sample size reduces the statistical power of the study, and thus, some important gene expression differences may not have been detected. Additionally, results from a limited cohort may not accurately reflect the wider population. To validate our observations and strengthen the conclusions, future studies with larger cohorts and more diverse patient samples are necessary. Secondly, although this study revealed mitochondrial dysfunction in epithelial cells, tissue stem cells, and macrophages as crucial in the development and progression of GDM by integrating RNA-*seq* and scRNA-*seq* data, the precise molecular mechanisms involved remain to be fully elucidated. Moreover, we did not assess immune cell infiltration in our mouse model through methods such as flow cytometry or multiplex IHC staining, which limits our ability to correlate scRNA-*seq* findings with experimental data. This analysis is important for understanding the interplay between mitochondrial dysfunction and the immune microenvironment, representing a significant area for future research. Furthermore, while we have confirmed the expression levels of hub genes in clinical samples and animal models, comprehensive *in vivo* and *in vitro* experiments are necessary to conclusively establish their roles in mitochondrial function and immune microenvironment homeostasis. Lastly, the potential application of the Mito-RGs score in non-invasive diagnostics has yet to be explored. Future studies should focus on evaluating whether biomarkers derived from the Mito-RGs score could be utilized in non-invasive diagnostic platforms, such as circulating extracellular vesicles or placental RNA, to enable early and precise prediction of GDM risk. This would advance the applicability of the findings to clinical practice and provide essential tools for optimizing GDM management.

## Conclusions

In conclusion, this study underscores the critical contributions of mitochondrial dysfunction and immune dysregulation to the pathogenesis of GDM. The identification of hub Mito-RGs (DHRS2, STX17, and TIMM44), alongside the development of a risk prediction tool (the Mito-RGs score-based nomogram), highlights the promising diagnostic potential and clinical utility. These findings provide valuable insights and a robust foundation for future clinical translation and precision medicine approaches to GDM.

## Data Availability

Publicly available datasets were analyzed in this study. This data can be found here: the GEO database at [ https://www.ncbi.nlm.nih.gov/geo, GSE203346, GSE173193].
