# Supplementary material for: Mitochondrial dysfunction and immune microenvironment in gestational diabetes mellitus: insights from bioinformatics analysis and experimental validation
Source: Front Immunol. 2026 Feb 26;17:1771616. doi: 10.3389/fimmu.2026.1771616 (PMC12979117; doi:10.3389/fimmu.2026.1771616)
Supplement: Supplementary file 1 [file DataSheet1.pdf]

Mitochondrial dysfunction and immune microenvironment in gestational diabetes mellitus: Insights from bioinformatics analysis and experimental validation

Running title: Mitochondrial dysfunction and immune infiltration in GDM

Rui Zhao [1], Tingting Chai [2], Qin Gao [3,*], Aimin Jiang [4,*]

[1] Department of Clinical Nutrition, The First Affiliated Hospital of Shandong First Medical University & Shandong Provincial Qianfoshan Hospital, Jinan, Shandong, PR China;

[2] Department of Obstetrics, Jinan Maternal and Child Health Care Hospital, Jinan, Shandong, PR China;

[3] Department of Public Health, Jining Medical University, Jining, Shandong, PR China;

[4] Shandong Provincial Key Laboratory of Precision Oncology, Shandong Cancer Hospital and Institute, Shandong First Medical University and Shandong Academy of Medical Sciences, Jinan, Shandong, PR China.

***Corresponding author:**

Aimin Jiang, M.D., Shandong Provincial Key Laboratory of Precision Oncology, Shandong Cancer Hospital and Institute, Shandong First Medical University and Shandong Academy of Medical Sciences, Jinan, Shandong, 250117, China; Email: jiangaimin199512@163.com;

Qin Gao, Ph.D., Department of Public Health, Jining Medical University, Jining, Shandong, 272000, PR China; Email: gqnutr@163.com.

Content

Table S1. Baseline characteristics of study participants in GSE203346.

Table S2. Baseline characteristics of study participants in GSE173193.

Table S3. Baseline characteristics of study participants.

Figure S1. Quality control of the GSE173193 single-cell RNA-seq dataset.

Figure S2. Ridge plots showing AUCell scores of pathway activity across major cell types in the GSE173193 single-cell dataset.

Figure S3. Representative immunohistochemistry (IHC) images and corresponding semi-quantitative IHC scores comparing Control and GDM placental tissues.

Table S1. Baseline characteristics of study participants in GSE203346.

Variables	Control (N=22)	GDM (N=21)	*P* value
Maternal age (years)	30.0 ± 3.2	31.0 ± 3.8	0.399
Gestational week (weeks)	39.0 ± 1.5	38.7 ± 1.1	0.402
Maternal weight (kg)	66.6 ± 5.9	73.6 ± 10.7	**0.013**
Maternal height (cm)	161.5 ± 5.0	162.3 ± 5.4	0.602
1-h PBG in OGTT (mmol/L)	7.3 ± 1.0	9.2 ± 2.1	**0.002**
2-h PBG in OGTT (mmol/L)	6.1 ± 1.0	7.5 ± 1.3	**<0.001**

Abbreviations: FBG, fasting blood glucose; GDM, gestational diabetes mellitus; OGTT, oral glucose tolerance test.

Values are expressed as mean ± standard deviation.

P values were obtained using *t* test for continuous variables.

Table S2. Baseline characteristics of study participants in GSE173193.

**Variables**	**GDM 1**	**GDM 2**	**Control 1**	**Control 2**
Age (years)	28	33	29	28
Height of delivery (cm)	150	163	162	164
Weight of delivery (kg)	62	83	64	61.5
Gestational age of delivery (weeks)	38+1	38+2	40+4	38+3
Nature conceived	Yes	Yes	Yes	Yes
Singleton pregnancy	Yes	Yes	Yes	Yes
Primigravid	Yes	Yes	Yes	Yes
Ethnicity	Han	Han	Han	Han
Cesarean section	Yes	Yes	Yes	Yes
Blood glucose of delivery (mmol/L)	3.53	5.16	4.36	4.39
OGTT (mmol/L)	4.46, 8.80, 8.7	5.1, 7.9, 9.5	3.73, 7.38, 7.02	4.54, 7.38, 7.02
Ultrasound abnormality	No	No	No	No
Medical diseases	No	No	No	No
Family genetic history	No	No	No	No

Abbreviation: GDM, gestational diabetes mellitus; OGTT, oral glucose tolerance test.

OGTT: oral glucose tolerance test,0h, 1h, 2h

Table S3. Baseline characteristics of study participants.

Variables	Total (n=12)	Control group (n=6)	GDM group (n=6)	*P* value
Maternal age (years)	29.73 ± 1.07	28.50 ± 2.88	30.33 ± 4.17	0.397
Height (cm)	162.00 ± 1.22	164.67± 5.16	161.00 ± 4.05	0.201
Pre-pregnancy weight (kg)	57.72 ± 3.16	52.83 ± 8.96	64.33 ± 8.93	0.050
Pre-pregnancy BMI (kg/m^2)	22.13 ± 1.44	19.44 ± 2.78	24.96 ± 4.43	**0.027**
Education level				
Less than high school	0 (0.0)	0 (0.0)	0 (0.0)	0.079
High school or equivalent	5 (41.7)	1 (16.7)	4 (66.7)	
College or above	7 (58.3)	5 (83.3)	2 (33.3)	
Primiparity				
No	6 (50.0)	3 (50.0)	3 (50.0)	1.000
Yes	6 (50.0)	3 (50.0)	3 (50.0)	
Gravidity (times)				
1	5 (41.7)	2 (33.3)	3 (50.0)	0.558
≥2	7 (58.3)	4 (66.7)	3 (50.0)	
FBG in OGTT (mmol/L)	4.81 ± 0.15	4.34 ± 0.24	5.20 ± 0.24	**<0.001**
1-h PBG in OGTT (mmol/L)	9.12 ± 0.48	7.68 ± 0.63	10.31 ± 1.01	**0.001**
2-h PBG in OGTT (mmol/L)	7.60 ± 0.60	5.96 ± 1.03	8.97 ± 1.45	**0.004**
SBP (mmHg)	112.91 ± 2.05	110.33 ± 4.93	115.17 ± 7.39	0.212
DBP (mmHg)	74.73 ± 2.38	75.83 ± 7.52	7500 ± 8.94	0.865

Abbreviation: BMI, body mass index; DBP, diastolic blood pressure; FBG, fasting blood glucose; GDM, gestational diabetes mellitus; OGTT, oral glucose tolerance test; SBP, systolic blood pressure; 1-h PBG, 1-hour post-load blood glucose; 2-h PBG, 1-hour post-load blood glucose.

Values are expressed as mean ± standard deviation or n (%).

P values were obtained using the chi-square test for categorical variables and *t* test for continuous variables.

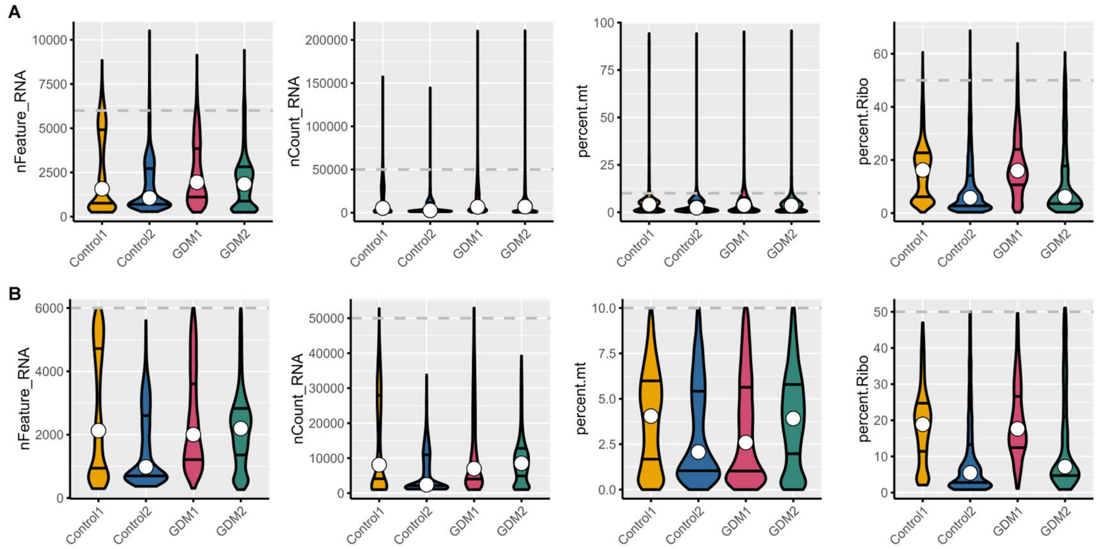

Figure S1. Quality control of the GSE173193 single-cell RNA-*seq* dataset. **(A)** Violin plots showing nFeature_RNA, nCount_RNA, percent.mt, and percent.ribo for each sample before quality control. **(B)** Violin plots showing nFeature_RNA, nCount_RNA, percent.mt, and percent.ribo for each sample after quality control.

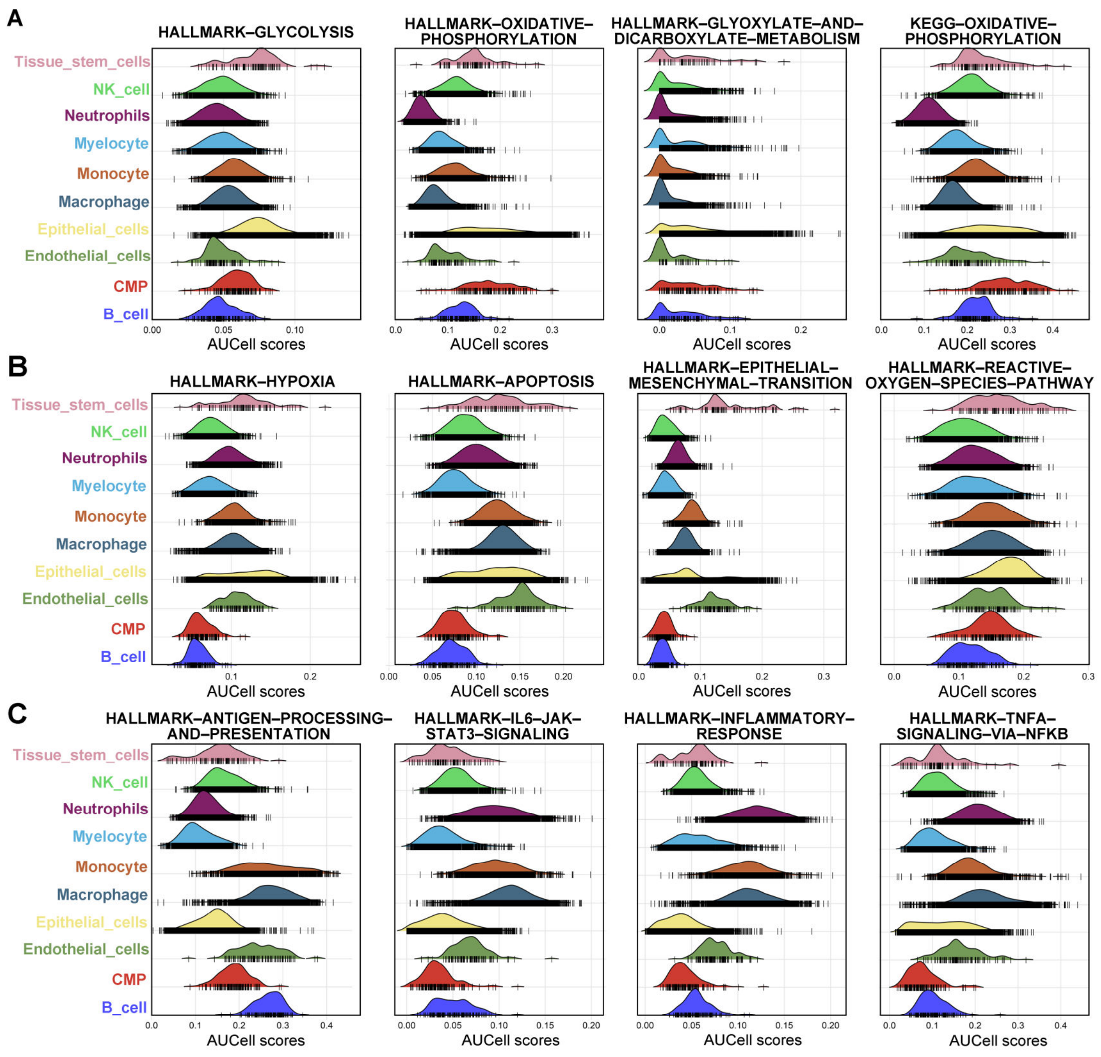

Figure S2. Ridge plots showing AUCell scores of pathway activity across major cell types in the GSE173193 single-cell dataset. **(A)** HALLMARK_GLYCOLYSIS, HALLMARK_OXIDATIVE_PHOSPHORYLATION, HALLMARK_GLYOXYLATE_AND_DICARBOXYLATE_METABOLISM, and KEGG_OXIDATIVE_PHOSPHORYLATION. **(B)** HALLMARK_HYPOXIA, HALLMARK_APOPTOSIS, HALLMARK_EPITHELIAL_MESENCHYMAL_TRANSITION, and HALLMARK_REACTIVE_OXYGEN_SPECIES_PATHWAY. **(C)** HALLMARK_ANTIGEN_PROCESSING_AND_PRESENTATION, HALLMARK_IL6_JAK_STAT3_SIGNALING, HALLMARK_INFLAMMATORY_RESPONSE, and HALLMARK_TNFA_SIGNALING_VIA_NFKB.

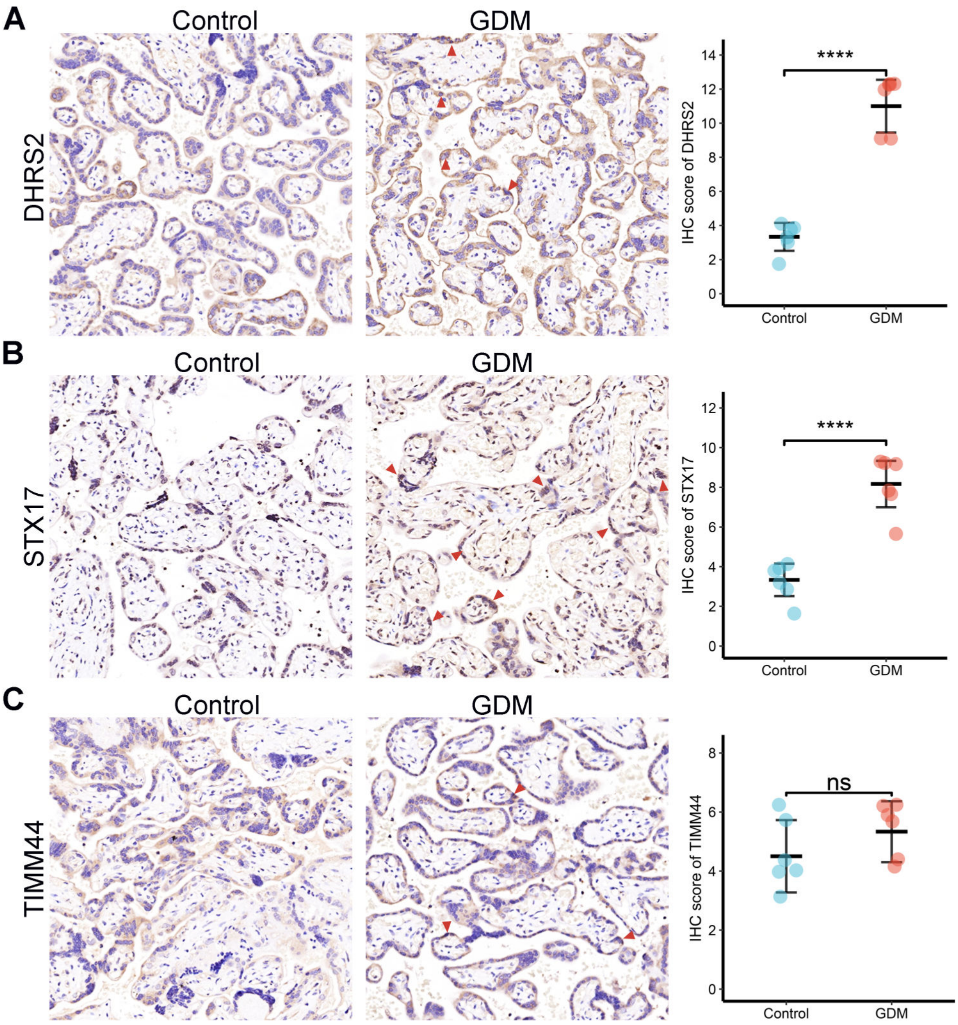

Figure S3. Representative immunohistochemistry (IHC) images and corresponding semi-quantitative IHC scores comparing Control and GDM placental tissues. **(A)** DHRS2 staining and IHC score. **(B)** STX17 staining and IHC score. **(C)** TIMM44 staining and IHC score. Points indicate individual samples; bars show mean ± SD. **** *P*<0.0001; ns, not significant.
